# Acceptability and feasibility of a behavioral and mobile health intervention (COMBIND) shown to increase uptake of prevention of mother to child transmission (PMTCT) care in India

**DOI:** 10.1186/s12889-020-08706-5

**Published:** 2020-05-24

**Authors:** Nishi Suryavanshi, Abhay Kadam, Savita Kanade, Nikhil Gupte, Amita Gupta, Robert Bollinger, Vidya Mave, Anita Shankar

**Affiliations:** 1 Lakshya Society for Public Health Education and Research, 307, Block II, Llyod Chambers, Mangalwar Peth, Pune, Maharashtra 411001 India; 2 Johns Hopkins University, School of Medicine, Baltimore, MD USA; 3 Johns Hopkins University, Bloomberg School of Public Health, Baltimore, MD USA

**Keywords:** PMTCT, HIV, Option B +, Intervention, Mhealth

## Abstract

**Background:**

A cluster-randomized trial recently demonstrated that an integrated behavioral and mobile technology intervention improved uptake of key components of a Prevention of Mother to Child Transmission (PMTCT) Option B+ program, among HIV- infected pregnant/breastfeeding women in India. To guide scale-up and optimize programmatic implementation, we conducted a mixed-methods evaluation of the feasibility and acceptability of this intervention.

**Methods:**

The COMmunity Home Based INDia (COMBIND) study, was conducted in four districts of Maharashtra, India and randomized 119 integrated counseling and testing centers (ICTC) and their outreach workers (ORWs) to the COMBIND intervention, an integrated mHealth application that allowed digital data capture, PMTCT educational videos, SMS alerts for missed visits and reminder for visits, combined with personal empowerment and motivational interviewing training for ORWs. This qualitative evaluation was done through 15 in-depth interviews (IDIs) with ORWs and 15 IDIs with HIV-infected pregnant/breastfeeding women from the intervention arm. Utilizing a concurrent nested mixed-method evaluation approach, we assess the feasibility and acceptability of the study intervention.

**Results:**

All 30 participants reported that the PMTCT videos were essential in providing easy to understand information on critical aspects of HIV and necessary care related to PMTCT practices. A majority of the ORWs reported that the personal empowerment training with motivational interviewing skills training increased their confidence, motivation and gave them the tools for effectively supporting their clients. The mHealth application improved their working style as it facilitated targeted PMTCT information support, systemized data capture, streamlined their health education delivery practice and provided a sense of work satisfaction. The SMS appointment alerts improved retention in HIV care for mother and baby to the smaller proportion that had access to their phones. Despite reported improvements in knowledge and communication, few ORWs reported that structural challenges such as limited drug stocks, lack of HIV kits or unavailability of trained staff at ICTC, may hamper the uptake of PMTCT services, thus resulting in limited significant impacts of COMBIND on PMTCT outcomes.

**Conclusion:**

This study found that COMBIND intervention is scalable, feasible, beneficial and very well accepted by ORWs and patients, however structural challenges in goods and services remain.

## Background

Important progress towards UNAIDS’ ambitious goal to end the AIDS epidemic in 2030 has been made, yet the prevention of new HIV infections remains a significant challenge [1]. Based on recent research trials, the most effective approaches include a combination of biomedical, behavioral and structural interventions to enhance uptake of prevention measures to reduce transmission of HIV [1–3]. The Government of India has pursued such efforts in its commitment to reduce HIV infections by 50% by 2020 [4]. Current estimates indicate that mother to infant transmission of HIV can reduce from 25% to < 2% if HIV-infected pregnant women start anti-retroviral therapy (ART) early in their pregnancy and maintain exclusive breastfeeding [5, 6]. These results helped inform the 2013 WHO revised guidelines that recommend the Option B+ approach and includes triple ART for all HIV infected pregnant and breastfeeding women, regardless of CD4+ cell count, intending to increase ART coverage and thus reducing vertical transmission [7]. This approach is designed to streamline the continuum of care related to the prevention of mother to child transmission (PMTCT), simplifying antiretroviral options to triple-drug therapy and avoiding the need for laboratory assessments to define ART eligibility or clinical disease staging. Based on these recommendations, India initiated the Option B+ roll out on January 1, 2014, which also includes the initiation of newborns on Nevirapine immediately after birth and promotion of exclusive breastfeeding.

Despite government efforts to make these services available in India, HIV-infected women still face numerous barriers to care and coverage remains a challenge. While 75% of pregnant women visited the antenatal clinic (ANC) at least once, only 40% completed three ANC visits. Also, only about half of the 14 million pregnant women targeted for HIV testing in 2016–17 completed testing [8]. Socio-economic factors including poverty, long distances to integrated counseling and testing centers (ICTC) and limited access to affordable transportation are major barriers to care. Moreover, the social stigma of HIV during pregnancy continues to play a role and can deter health-seeking [9, 10]. While substantial research on the effectiveness of PMTCT has been done in many African countries, limited research has been conducted in India.

Mobile technologies have been advocated as an important tool to support behavior change by improving both access to and quality of health care delivery in resource-poor settings [11–14]. Mobile-based interventions can provide consistency in the delivery of an intervention and the ability to disseminate the intervention to a wider population [15] leading UNAIDS to recommend that mHealth approaches be incorporated into national HIV care programs where possible.

However, technological interventions still rely on the competency and capacity of the local health service delivery persons to effectively be a bridge between medical guidelines and patients needs. Most technological interventions focus on supplying accurate health information, rarely addressing challenges faced by health workers themselves in effectively communicating with patients. Moreover, in the case of HIV, many ORWs are from similar communities, are HIV -infected and often experience the stigma, poor health and life challenges faced by the patients they serve. The COMmunity Home Based INDia (COMBIND) intervention examined in this paper, provided a cognitive-behavioral training to ORWs designed to increase their motivation, improve communication skills and enhance their personal agency to improve ORWs capacity to support the needs of their HIV-infected pregnant and breastfeeding clients. Details of evaluation of the main trial are reported elsewhere. Using a concurrent, nested, mixed-methods design, this paper reports on the qualitative results of the impacts of an integrated mobile health and behavioral intervention that aimed to enhance the capacity of outreach workers (ORW) in the national program, to optimize PMTCT, Option B+ services uptake.

## Methods

### Study setting and primary study

**Fig. 1:**
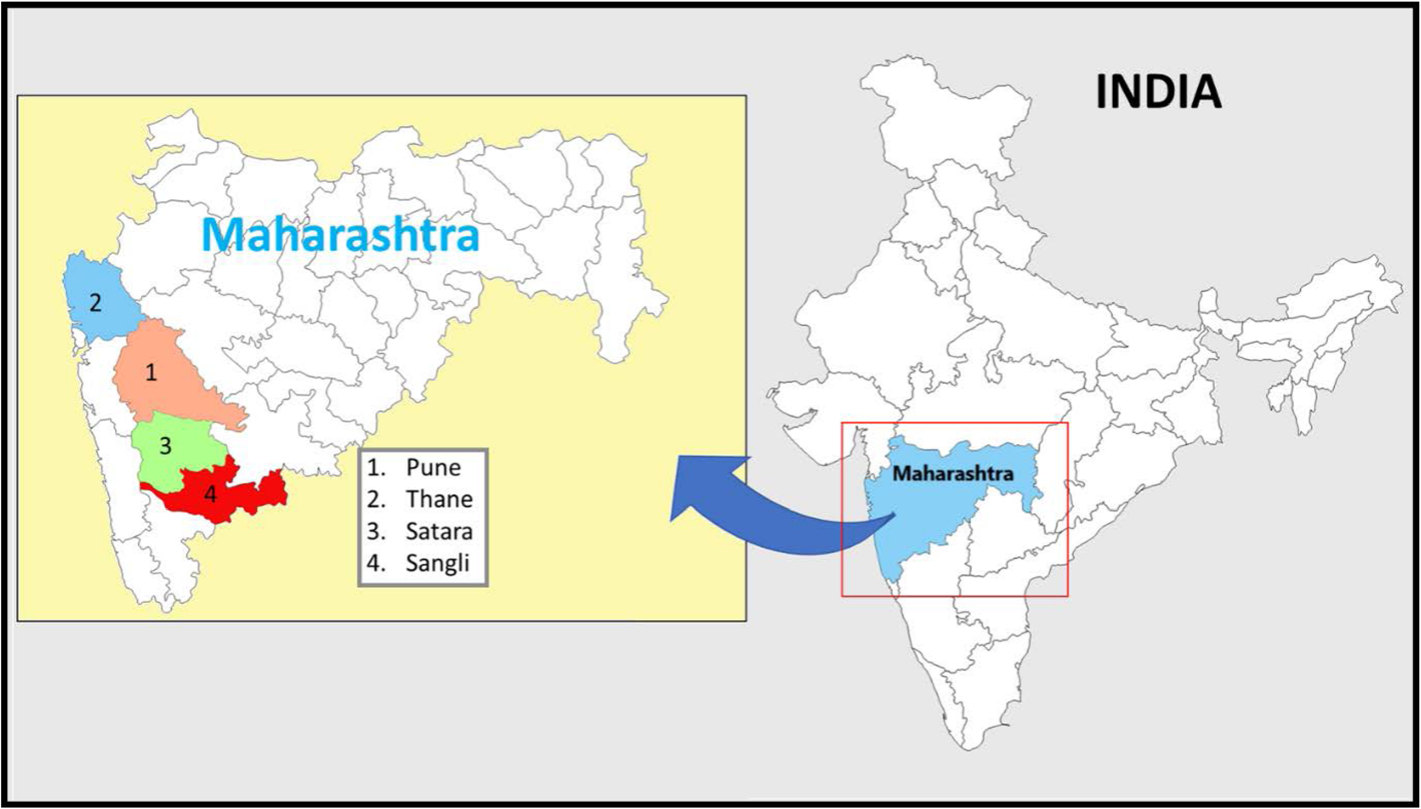
Study districts in Maharashtra state, India. Four high HIV burden study districts Sangli, Satara, Pune and Thane in Maharashtra state in India. This Map is prepared by AK using MS office (MS word and PowerPoint) tools

The primary study was conducted in four high burden districts of Maharashtra (Pune, Sangli, Satara, and Thane), one of the highest HIV prevalence states of India (Fig. 1).

The COMBIND study was a cluster-randomized trial conducted from April 2015–March 2017 in 119 ICTCs randomized to either standard of care or intervention and included an integrated mobile health and behavioral training intervention for ORWs at the ICTC. HIV- infected pregnant and breastfeeding women’s participation in the PMTCT Option B+ was evaluated over 18 months. The concurrent, nested qualitative component of this mixed-method approach provides insights related to the integrated mobile health and behavioral intervention from the perceptive of ORWs and HIV-infected pregnant and breastfeeding women.

This study was built upon the existing efforts of India’s National AIDS Control Organization (NACO) phased-in implementation of the 2010 WHO revised PMTCT guidelines for universal provision of ART to HIV-infected pregnant and lactating mothers, six months exclusive breastfeeding (EBF), six weeks of infant nevirapine prophylaxis and early infant diagnosis (EID).

NACO, through State AIDS Prevention and Control Societies (SACS) and nationally appointed local non-governmental organizations (NGOs), engaged ORWs for community mobilization to access PMTCT services who received HIV training with particular focus on PMTCT services. ORWs made monthly home visits to the HIV+ pregnant woman during pregnancy, labor and immediately afterward as well as post-delivery, up to 18 months to optimize the uptake of PMTCT services. Additionally, COMBIND intervention provided ORWs with integrated mhealth and behavioral training based on sIMB framework [16] to efficiently engage with their clients to increase the uptake of PMTCT services.

### COMBIND intervention

In India, despite the fact that 97% of HIV-infected pregnant women and their babies received antiretroviral prophylaxis for PMTCT [17], poor outcomes persist due to challenges at personal, social, and structural levels [18]. The critical need to design and implement effective interventions to overcome these contextual barriers and increase the uptake of PMTCT services has been well reported [19–21]. A technology-based behavioral intervention COMBIND was developed to help ORWs link their clients to PMTCT services more efficiently to provide uniform messaging and address gaps in care.

The COMBIND PMTCT intervention included a mHealth platform EMOCHA [22] (Electronic Mobile Comprehensive Health Application) that allowed the use of tailored “smart forms” (i.e. forms that can respond automatically with pre-programmed algorithms) to strategically and efficiently guide ORWs, as a clinical decision support system. The ORWs visited pregnant/ breastfeeding women in their homes providing them with information on care for PMTCT through the use of videos, collected information using smart forms and sent SMS alerts for upcoming visits/missed visits. Four specially designed videos were created on 1) exclusive breastfeeding, 2) how to take ART medicines, 3) disclosure and 4) ensuring HIV testing of babies. Each video was available in both Marathi and Hindi. Videos can be found here (www.https//Lakshya.trust.co.in). In addition to the mHealth component, a one-week residential behavioral training course was designed to assist ORW’s engagement with HIV-infected women. This training included personal empowerment to enhance ORWs self-awareness, increasing their motivation and confidence for the work [23]. Training on motivational interviewing to support the HIV infected pregnant and breastfeeding women’s improved health-seeking behaviors was also included [24]. In addition to the residential training, a three-month refresher training was conducted.

### Data collection

#### Quantitative methods and analysis

The four primary study outcomes included: 1) proportion of HIV+ women on ART at delivery; 2) proportion of HIV-exposed breastfed infants provided six to 12 weeks of Nevirapine; 3) proportion of infants receiving EBF at 6 months, and 4) proportion of HIV-exposed infants screened for HIV infection at six months of age. Secondary study outcomes included EBF at six weeks, EID at six weeks, 12 months and 18 months, missed EID visits, and maternal and infant mortality. Detailed methods and quantitative results are explained elsewhere (forthcoming).

#### Qualitative methods and analysis

The qualitative evaluation focused on understanding ORWs and HIV-infected pregnant and breastfeeding women’s perceptions of the effectiveness and utility of the mHealth and behavioral intervention. Qualitative data is reported as per consolidated criteria for reporting qualitative studies [25] and included 15 in-depth interviews among ORWs who were randomly selected from the pool of total ORWs (*n* = 60) from the intervention arm, stratified by district. A similar process was used to identify 15 HIV- infected pregnant and breastfeeding women. The in-depth interviews were guided by an ethnographic field guide designed separately for the ORWs and patients. For ORWs, the interview guide was designed to elicit experience with, and perceptions of, study videos, SMS alerts, change in personal motivation and communication skills, interactions and reflections of patient uptake of PMTCT services, the overall intervention deployment process and intervention’s potential to scale (Additional file 1). For patients, the interview guide focused on experience with the COMBIND intervention, such as videos and SMS alerts, as well as patient’s experience with engaging with the ORW and challenges faced in health-seeking (Additional file 2). The interview guides were pre-tested and validated through focus group discussions with ORWs and patients [18].

**Fig. 2:**
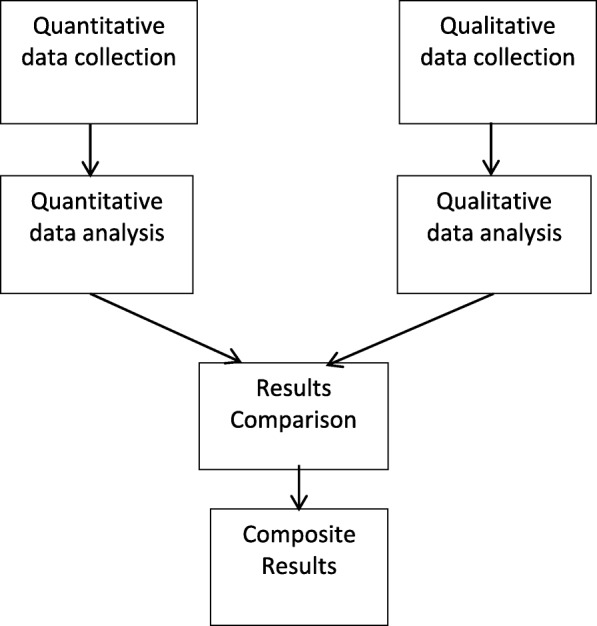
Concurrent Triangulation of data for Mixed Methods evaluation. Triangulation of quantitative and qualitative data is done to present the composite results for acceptability and feasibility of COMBIND intervention

Written informed consent was obtained from all participants and interviews were conducted either at the respondent’s home, the ICTC or at the NGO offices as per the respondent’s convenience to maintain complete privacy. Each interview lasted between forty-five minutes to one hour. Interviews were conducted by two female social scientists who were trained by NS, AK, and SK. All participants were approached by ORWs and study counselors through face to face meetings and introduce social scientist to participants. Interviews were tape-recorded, transcribed and translated into English by trained behavioral scientists and were audited for accuracy by NS and AK. Qualitative content analysis was conducted using the Framework Approach [26] that systematically categorizes and organizes data allowing for identification of commonalities and differences in text-based data, resulting in descriptive and/or explanatory conclusions clustered around major themes. Completed transcripts were coded by NS, AK and AS using a system that elucidated recurring themes and ideas identified in the initial formative research phase with input from the team behavioral scientists and interviewers. Samples of coded text were reviewed regularly among the research team to further delimit theme definitions and check for coder reliability. Any discrepancies in coding were discussed until consensus was achieved. The codebook was revised as new themes emerged, either by creating new codes or sub-codes under existing themes. Associations between themes were also identified using MAXQDA software. Findings from both methods were combined to provide a holistic view of the research question being examined (Fig. 2).

## Results

### Quantitative findings

The outcomes of the COMBIND study are reported elsewhere (citation masked for peer review); however, for this mixed-method evaluation in the context of the main trial, a summary of the main trial findings (quantitative results) are presented below. The COMBIND study focused on the impact of the mHealth and behavioral intervention on uptake of the proportion of HIV infected pregnant and breast-feeding women on ART at delivery, proportion of HIV-exposed infants on nevirapine prophylaxis, exclusive breastfeeding at two and six months and receiving early infant diagnosis at six weeks and six months. Overall uptake of key PMTCT services was high in both COMBIND PMTCT and standard of care (SOC) arms. There were no statistical differences between the COMBIND and SOC arms for ART at delivery, EBF at 6 months, infant nevirapine or EID at 6 months. The COMBIND intervention did, however, show significantly increased uptake of EBF at 2 months (aOR, 2.10; 95% CI 1.06–4.15), and EID at 6 weeks (aOR, 2.19; 95% CI 1.05–3.98) demonstrating that the COMBIND intervention increased early uptake of EBF and EID among HIV-infected pregnant and breastfeeding women.

### Sample

Characteristics of the 15 ORWs are as follows. All 15 ORWs interviewed were females, with an average age of 39 years. Nine were widowed or separated, 11 (73%) had at least a secondary level education and all but one has been tested for HIV, of which 7 (47%) were HIV infected and on ART. Of the 15 HIV infected pregnant and breastfeeding women who were interviewed, all were married, with an average age of 23 years. Fifty percent of them did not have any living child and 9 (60%) had completed a secondary education.

### Qualitative findings

#### Response of ORWs to intervention

Perceptions about the intervention were categorized into 4 major themes; 1) the mobile health platform helped ORWs effectively do their job; 2) the behavioral training increased self-awareness and improved relational skills 3) the combined intervention supported greater uptake of four key PMTCT components, however, structural constraints persist and 4) the intervention can be optimized and scaled for India.

1. The mobile health platform helped ORWs effectively do their job:

All of the ORWs reported satisfaction with the EMOCHA mHealth application, despite some initial apprehension in using the tablets. Generally, the ORWs found the message prompts critical in helping them collect data correctly and reminding them of the information to be conveyed on key PMTCT components. There were minimal issues related to network connectivity and sending out forms. Another, more contextual issue was whether the patient had disclosed her status with other household members, which required moving the meeting to a more private location, which was easily resolved. Overall, there were rarely challenges in using the tablet. The videos were a primary component of the mHealth support, however, their utility was limited to a single showing as most clients expressed a desire not to see them repeatedly. Patients appeared more comfortable to view the videos at the ART center rather than within their home. The ORWs found themselves needing to balance the utility of the information from the videos with the likelihood that the patient may feel bad when she is reminded of her medical condition. The ORWs felt the videos were useful to the clients and the information was presented in an easy to understand format. Nearly all ORWs mentioned that the videos helped increase the client’s understanding of their condition and best practices for themselves and their children. Having videos in multiple languages was key in enhancing comprehension.

The ORWs indicated that the videos reduced the time needed to talk about these issues. The videos also reinforced content that the ORW may not have been clear about. One remark from an ORW was:

“*…*. I*nstead of speaking constantly with them, clients can understand more through the videos, they get all the answers for their questions which they want to ask us before the visit or whenever they ask me anything that time I tell them … “I know all the answers for your questions but first, you should watch the video so that you will get more information than me speaking to you.” We are also unaware of some topics like if the baby vomits while taking medicine then what we should do? Literally, I didn’t know about this but after watching that video I could also get the knowledge about that so if I carefully show the videos to my clients, it would be understandable to them and some clients are very good at adapting … things which are shown in the video … questions are quickly answered …*” ***IDI#11_O0480482***

2. The behavioral training increased self-awareness and improved relational skills

The personal empowerment training component, which was a central part of overall behavioral intervention appeared to boost ORWs confidence and helped them improve their communication with their patients. Nearly all ORWs mentioned that they felt that the patients were now empowered enough to make their own decisions after the ORWs have worked with them. Several ORWs reported that the personal empowerment training combined with training on motivational interviewing skills gave them both the confidence and the tools for effectively supporting their patients. ORWs mentioned that patient’s brought up appointments, taking medications or diagnostic tests without being prompted. As mentioned by one ORW:

*“We have changed our way of speaking. Training or whatever videos we have shown to them maybe those are helpful therefore clients are getting improved. Firstly, we have changed and due to our change, there must be some change in clients as well … means they should not have to depend on us for their work but they should be able to do their work on their own.”****IDI#05_O0880881_14.12.2016.***

One ORW mentioned that because they became more skilled in discussing issues and guiding them, patients were taking more initiative. An example is related to financial support. In the past, many ORWs reported giving their own money to cover the costs of travel for their patients. But now, after explaining what is being provided by the government related to the costs of ART, etc., the patients began to understand the value of what they are getting and then find ways to pay for travel costs. A few ORWs mentioned that while patients are, in general, improving their ability to take initiative, there are always a few patients that are not able to comply. ORWs felt that sometimes that was due to their social situation (e.g. husband doesn’t agree) or lack of financial resources. However, even in these cases the ORW reported seeing improvements and continued to foster the patient’s empowerment.

3. The combined intervention supported greater uptake of four key PMTCT components, however, structural constraints persist

i. Improved initiation and adherence to ART:

ORWs shared that after using study videos and counseling skills they learned in empowerment training, patients improved their adherence to ART. Five ORWs reported that many patients are taking ART regularly except a few patients who are discouraged by side effects they experienced. Due to pressure from hospital staff, these patients collect medicines regularly, however, they may not consume them. For these cases, several ORWs shared that using counseling skills, study videos, and their empowerment skills they were able to successfully change the behavior of a few of such patients. For example, as reported by one ORW:

*“I showed her two videos … at once … her expressions were good, when she watched those videos & after watching it she said, “I was not informed like this at ART center, I’m listening to this information for the first time from you” then after on next day she came with her husband for taking ART”.***IDI#11_O0480482.**

Nearly half of the ORWs (*n* = 7) reported that patients experiencing side effects and perceptions about potential side effects influenced ART adherence. A similar number reported issues related to the health system, such as the negative behavior of clinic staff, long distances to the clinic, lack of an accompanying person, shortage of drugs, limited counseling by hospital staff, problems in transferring patients to link ART centers (which are closer to patient’s residence) before 18 months (baby’s) influenced ART adherence.

ii. Videos helped address misinformation on infant feeding

Seven ORWs reported that study videos helped them tackle the issue of conflicting advice patients received from counselors, ORWs and doctors on exclusive breastfeeding. They shared many success stories on how they tackled this kind of issue using study videos, counseling, creating confidence in patients and convincing doctors as well as patients about the benefits of exclusive breastfeeding. As indicated by one ORW:

*“There was considerable difficulty … doctors in the Civil hospital (government-run hospital) used to advise about top feeding (giving diluted animal milks) and we were informed about breastfeeding, so women in the village used to give mixed feeding and are not listening to us. Previously they were breastfeeding as well as top feeding then we counsel them saying, “New guidelines have come regarding breastfeeding and we should do according to those guidelines only since powder milk is not always available to us and before giving milk (to baby) we have to take care about the preparation of that milk as well as we have to boil that container in which we poured the milk so instead of doing a lot of this it is better to give mothers milk and you are taking your medicines regularly so there is not at all chance to transmit this infection through you to the baby” we are explaining like this and along with this we are showing our videos to them so, now they are listening to us”.***IDI#09_O0160164**

iii. While infant prophylaxis was generally good, the intervention supported greater ORW motivation

Giving infant prophylaxis did not seem to be an issue, except for the few cases of home deliveries, especially if the births occurred on the weekend. Six ORWs described their support of patients to get infant prophylaxis. In rural areas, ORWs made sure that patients collect an extra bottle of infant prophylaxis at the time of their ART medicine visit and guide illiterate patients. In this case, the ORW demonstrated significant motivation to assist the patient to prevent PMTCT.

*“One home delivery was there so I took Nevirapine to her home and gave to the baby. Her husband had called and told me “she is in labor pain, what I do?” I said to him that admit her into the hospital but until she sits in the vehicle she got delivered and her mother-in-law did everything for her after delivery and there was a holiday on next day but, I went to the ICTC, took Nevirapine, went to her home and gave Nevirapine to the baby and provided all the instructions regarding Nevirapine”.***IDI#06_O01330243**

iv. Supply-chain issues and reluctance for multiple testing hampered getting infants tested for HIV

Eleven of 15 ORWs reported systems-related issues that hampered uptake of PMTCT services such as lack of HIV kits and trained staff at ICTC clinic for babies testing. If an infant had an earlier negative HIV test, parents are also reluctant to retest their child. As one ORW mentioned:

*“As I told you due to the technician’s marriage six months testing was not done for one of my patient’s babies. Many times, patients are not doing the second test (6 months testing) if the first test will have a good report (negative); they are thinking, if the first test is negative good then all other tests will be good. Actually, always we are convincing them about the necessity of testing four times. But people from our side (hospital staff) are not that serious about this which makes it more difficult for us to convince them to do more frequent testing”.****IDI #04_*O0910911.**

4. The intervention can be optimized and scaled for India

Suggestions to optimize this intervention included the provision of headphones to increase confidentiality concerning video content. Having videos on family planning methods during sexual contacts to avoid pregnancy would be helpful as well as videos directly addressing how to deal with the stress and fear associated with learning about their diagnosis. Also, ORWs felt that videos that described what was learned in the personal empowerment training would be helpful*.* As mentioned by an ORW:

*“You are giving very good services … .everything is going to be proper here … .but if some changes are there then it will be good (Interviewer: What we can do?) … we are showing videos to them that is a good thing but we should have something like that in their language for their positive thinking and it is necessary because every client is not educated hence we should have to make such kind of video also in which we can give lessons about empowerment and independence for illiterate women also..”****IDI#14_O520522***

i) Content on tablets are easy to use and be understood

ORWs mentioned the ease of using tablets instead of mobile phones and how it benefited patients. More than 50% mentioned that tablet is feasible and acceptable to show videos and to capture data. Tablets have big screens making it easier to show the videos and likely to be understood by the patients. Videos allowed patients to understand the concepts more clearly and they felt that this could be easily rolled out at the national level. ORWs felt it would not be difficult if proper training is given to them and data is captured in the local language. Some ORWs reported this intervention resulted in a major change in their working style and they found data capture was more systematic and easier. ORWs reported that continual education is supported through the use of the tablet and suggested that a range of health care workers, such as ASHAs (Accredited Social Health Activists) and Anganwadi (focused on women and child health and nutrition) workers could easily be trained in this type of intervention. That would streamline their work and staff at ANC, ART and ICTC centers would have access to patient’s information directly.

#### Response of HIV infected pregnant and breastfeeding women to intervention

Women’s perceptions about the intervention were categorized into 3 major themes: 1) women understood the content of PMTCT videos; 2) SMS alerts were useful, but access was at times challenging, and 3) positive feedback about ORW’s work in the intervention arm.

1. Women understood the content of PMTCT videos

All participants reported watching videos and felt the information from these videos was supported by explanations given by the ORWs. Most notable was that patients remembered the important topics from the 4 study videos; breastfeeding, how to take ART medicines, about disclosure, and regular HIV testing of babies. This is reflected in the quantitative results showing that exclusive breastfeeding uptake increased in the intervention arm. The overall feedback from participants indicated they liked watching the videos as it clarified several concepts for them. Many indicated they had never been given this information earlier.

2. SMS alerts were useful, but access was at times challenging

Nine patients provided feedback on the utility of SMS reminders. Of the sample of 15 women, two-thirds [10] reported challenges in access; two had no phones, one was illiterate so did not see any messages and seven of the patients reported that they did not have their phone but gave their husbands phone numbers. Only two said that their husband used to tell them regularly about reminder messages about the visits/ missed visits. As mentioned by one patient:

“*The mobile phone is with my husband hence I am not aware of those messages but before any message, I would have remember every date testing and ART also and I do planning according to that date … ..may message be coming but my husband ignore those … ..maybe the message came on my old mobile also but currently that mobile is not working”.***IDI#12_P1512.**

The remaining five that had access to phones reported that it helped them remember the exact date of their and their babies visit. All five patients mentioned how the SMS component of the intervention improved their adherence as it is used to convey reminders to them that was easy for them.

*“Yes... It is helpful. Like we get a reminder that if my husband is not there then at least I can go … as it had happened one time, when I was pregnant, my husband was in Pune so due to the message only, I understood that I have to go for medicines. So during that time, I went alone”. IDI#15_P0612.*

3. Positive feedback about ORWs work

Almost all patients reported the strong support they received from ORWs and how they provided information on how to take care of themselves and their children. All patients perceived ORWs as a great source of support and information. When asked whether they will be able to continue PMTCT care and ART care if the ORW were not there, many patients mentioned that ORWs are required to support them to provide the information and to help them with all PMTCT service access.

*“If she would not be there then, many patients would not know how to take care of babies. They will think that as we have this disease, our baby will also get it … baby will be positive. But didi (ORW) would meet these people and give them information … they will not lose hope then … I have seen such people … but today I can myself assured them and give them correct information … If people do not get information, how they would know what is correct and what is wrong? They would only think that now due to this disease our life is ruined … everything is destroyed … and because of us our baby’s life will be ruined. All the questions that I ask she answers. She is there with me whenever I need her”.****IDI #14_P0532.***

## Discussion

The qualitative data from this mixed-methods evaluation of the COMBIND intervention study indicates that an integrated mHealth and behavioral training intervention is a feasible and acceptable approach for the delivery of comprehensive PMTCT information in low resource settings. Both the mHealth and behavioral training were well accepted by the ORWs and the specific digital content appears to successfully convey information to patients on the option B+ guidelines. These findings are relevant for other programs that are attempting to promote preventive health behaviors along the continuum of care. The COMBIND integrated intervention addresses key challenges identified in previous research reviews [27–29]. Earlier formative research [18] highlighted the need to provide accurate up-to-date information on the latest PMTCT services and also stressed the need to support the personal challenges faced by ORWs to effectively do their job. The COMBIND intervention attempted to address these challenges with a combination of 1) a specialized behavioral training for the ORWs; 2) content specific counseling and educational scripts and videos; 3) a tablet-based mobile health application designed to support data collection and 4) SMS appointment alerts for patients and ORWs.

Based on the qualitative interviews of the ORWs and patients, the integrated intervention was reported to be highly effective and impactful in facilitating the interactions between the ORW and patient. ORWs found the tablet and EMOCHA program easy to use and the content relevant for their patients. There was some evidence that clients preferred to watch videos in private spaces, where confidentiality could be maintained and showing more than one video at a time appears to be strenuous and/or not interesting to the clients.

Overall, the increased ORW skills combined with topic-specific videos and counseling scripts were perceived to be effective to support all four key PMTCT services, however, uptake of only two, early infant diagnosis at 6 weeks and exclusive breast-feeding showed significantly higher in COMBIND arm in the quantitative analysis. While our findings share some similarities with earlier studies [30, 31] suggesting that behavioral or psychological interventions may influence components of the PMTCT cascade. In the case of early infant diagnosis at month 6, we identified other critical factors confounding uptake, such as lack of HIV kits and properly trained ICTC staff, and reluctance of parents to continually retest their child after an initial negative test result. The structural challenges are not easily overcome through an improved counseling process. Moreover, most health staff do not stress the need for multiple testing for infants, which is further complicated by the parent’s reluctance to continue to obtain tests after the child has tested negative.

There is an indication that the intervention was supportive of better care-seeking. In the case of adherence to ART, the information from the videos appeared to convince some patients to be more diligent in continuing to maintain life-long adherence. Similarly, with exclusive breastfeeding, patients appear more confident to continue breastfeeding after the video and counseling. To supporting infant prophylaxis, the majority of women had a hospital or clinic deliveries and therefore obtained the appropriate treatment for their infants. Our qualitative results reveal that ORWs showed greater motivation to support the few patients that had home deliveries by ensuring their patients had sufficient drugs for their child at delivery.

The utility of the SMS as appointment reminders did not appear to optimally support this population. This may be due, in part, to the limited access that patients have to their mobile phones. These data reinforce the idea that while SMS messaging may be desired by clients to help remind them of appointments [32, 33], the realities of limited phone access appears to reduce the overall applicability of SMS for vulnerable populations. More effective male partner involvement could potentially enhance the COMBIND program, although limited data are available on the effectiveness of such interventions [34].

The qualitative results from patients emphasized the critical role that ORWs play in the life of HIV-infected women in India. Overwhelmingly, patients reported how ORWs provided important information, social support and guidance for the women and her family. Having ORWs that are confident about their knowledge and ability to effectively engage and counsel their clients are more likely to continue to provide a high level of care to their clients.

A recent systematic review of interventions to improve PMTCT [35] identified only a handful of studies that detailed infant outcomes, only three randomized trials and only one mHealth intervention. Authors suggest that interventions that included ART integration and the use of lay providers would be likely to increase service uptake [35]. Similarly, a mapping of interventions to address PMTCT in Sub-Saharan Africa [36] suggests the need for multifaceted approaches that respond to the contextual settings, a primary emphasis of COMBIND. The site and topic-specific videos and scripts, as well as appointment reminders, enabled ORWs to better target support for their clients. Strengthening ORWs confidence, self-esteem and capacity, served as a bridge between technology and the client. Based on the experience of both the ORWs and patients, the COMBIND intervention can effectively support this level of care, which can be easily adapted for scale.

## Conclusion

This integrated mHealth and behavioral intervention was successfully implemented in four high HIV burden districts of Maharashtra. While quantitative study results could show a significant impact of the intervention on only two of the secondary outcomes, the qualitative evaluation demonstrated considerable benefit and support for intervention by ORWs and patients in enhancing uptake of PMTCT services.

## Supplementary information

**Additional file 1.**

**Additional file 2.**

## Data Availability

The datasets analyzed during the current study are available from the corresponding author on reasonable request.

## Abbreviations

PMTCT: Prevention of Mother to Child Transmission
COMBIND: COMmunity Home Based INDia
ICTC: Integrated counseling and testing centers
ORWs: Outreach workers
IDIs: In-depth interviews
ANC: Antenatal clinic
NACO: National AIDS Control Organization
EBF: Exclusive breastfeeding
EID: Early infant diagnosis
SACS: State AIDS Prevention and Control Societies
NGOs: Non-governmental organizations
EMOCHA: Electronic Mobile Comprehensive Health Application
SOC: Standard of care
ASHAs: Accredited Social Health Activists
