# Supplementary material for: Acceptability and feasibility of a behavioral and mobile health intervention (COMBIND) shown to increase uptake of prevention of mother to child transmission (PMTCT) care in India
Source: BMC Public Health. 2020 May 24;20:752. doi: 10.1186/s12889-020-08706-5 (PMC7245843; doi:10.1186/s12889-020-08706-5)
Supplement: Supplementary file 1 — Additional file 1. [file 12889_2020_8706_MOESM1_ESM.pdf]

In-depth Interview guide (ORWs: Intervention arm)

Welcome. Thank you for coming. As was mentioned during recruitment, we would like you to participate in an interview. Today, we are going to talk about your knowledge and experience with this intervention (emocha platform, empowerment and motivational interviewing training) and about the implementation and scale up of this intervention and challenges you faced. As discussed in the consent form, you do not have to answer a question if you do not want to. Now, let's start our discussion.

- Tell me a little about your typical day/activities of imparting PMTCT services. What do you do?
- What are your views of the emocha and motivational interviewing training in enhancing your knowledge, interviewing skills, and counselling skills?
- What has your experience been in implementing the interviewing and counseling techniques?
- What are your views on the feedback system and the additional counselling training? Has it improved your motivation in your work?
- What was your experience with emocha to implement Option B+ PMTCT services? Please explain. (Probes: Advantages, Challenges with each component, ART initiation, NVP prophylaxis, exclusive breastfeeding, EID testing).
- What are your view on this intervention (your training in motivational interviewing technique and emocha) increasing uptake of PMTCT services? (Probes: Advantages, Challenges).
- What challenges did you face in the community and in the ART clinic to complete smart forms on the phones/tablets?
- What are your views on scaling up emocha/behavioural intervention to the national level?

Conclusion

Thank you very much for participating today. I have learned a lot from you. I appreciate your openness and I will maintain confidentiality of our conversation as discussed in the consent form.
