# Supplementary material for: Acceptability and feasibility of a behavioral and mobile health intervention (COMBIND) shown to increase uptake of prevention of mother to child transmission (PMTCT) care in India
Source: BMC Public Health. 2020 May 24;20:752. doi: 10.1186/s12889-020-08706-5 (PMC7245843; doi:10.1186/s12889-020-08706-5)
Supplement: Supplementary file 2 — Additional file 2. [file 12889_2020_8706_MOESM2_ESM.pdf]

In-depth Interview HIV+ Women

In-depth Interview guide (Pregnant/breastfeeding women: Intervention arm)

Welcome. Thank you for coming. we are planning to have an interview that we would like you to participate in. Today, we are going to talk about your knowledge and experience with your ORW giving you information about preventing HIV transmission to your baby using a tablet/phone. You do not have to answer a question if you do not want to. Now, let's start our discussion.

Please tell me about your home life?

- Who do you live with? (Ask about family members and how they start their typical day)
- To what extent are you free to make your own choices about issues in your life such as pregnancy/HIV care and treatment?
- Has the ORW positively influenced your life? If so, how?
- If you have any personal problems, who do you go to? (Probe for health problem, family problems and any other problems due to HIV)
- What are your views on the ORW meeting your expectations in giving PMTCT care and counselling?
- What are the benefits of PMTCT services?
- Have you ever heard about HIV prevention to your baby before your pregnancy?
- Where did you get this information? (probe about how much each source of information is trusted)
- How did your ORW approach you? (how did she introduce herself to you or to your family when she is visiting you at home)?
- How did you find your ORW giving you information through videos and collecting information from you? What are your views on using video to educate the community? (Probe for acceptability and feasibility)
- What kind of challenges did you face to exclusively breastfeed your baby for 6 months? Did you get any help? Please explain.
- Where do you get your ART? What information ORW provided?
- Do you face any problems in getting your ART medicines? If so, please explain.
- Did you face any problems getting daily infant prophylaxis for your baby? If so, please explain.
- When you visit the ART clinic for your appointments (pick up medication/CD4 count/EID etc.) do you face any challenges? If so, please explain.
- What do you think of videos you have seen on ART, Exclusive breastfeeding, EID and giving NVP to your baby?
- How you liked receiving feedback and visit reminders on your phone?
- Would you like your ORW to improve on some things? If so, what should your ORW improve on?

Conclusion

Thank you very much for participating today. I have learned a lot from you. I appreciate your openness and I will maintain confidentiality of our conversation as discussed in the consent form. Thank you again.
